# Commentary – ordering lab tests for suspected rheumatic disease

**DOI:** 10.1186/1546-0096-6-19

**Published:** 2008-11-17

**Authors:** James N Jarvis

**Affiliations:** 1 Department of Pediatrics, Rheumatology Section, University of Oklahoma College of Medicine, Oklahoma City, OK 73104, USA

## Abstract

One of the least-appreciated advances in pediatric rheumatology over the past 25 years has been the delineation of the many ways in which children with rheumatic disease differ from adults with the same illnesses. Furthermore, we are now learning that paradigms that are useful in evaluating adults with musculoskeletal complaints have limited utility in children. Nowhere is that more true than in the use of commonly used laboratory tests, particularly antinuclear antibody (ANA) and rheumatoid factor (RF) assays. This short review will provide the practitioner with the evidence base that supports a more limited use of ANA and RF testing in children.

## Commentary

You've probably heard this dozens of times before, "This might be a collagen vascular disease. Order an ANA and a rheumatoid factor." It's common on adult wards, but it's used with disturbing frequency on pediatric wards as well. One of the least appreciated advances in pediatric rheumatology over the past 20 years has been the realization that models used to evaluate adults with musculoskeletal complaints do not serve children well [1]. Thus, it is perhaps no surprise that children with even the most common form of juvenile idiopathic arthritis (JIA- the currently accepted designation for the forms of chronic arthritis that include what was previously termed juvenile *rheumatoid *arthritis, J*R*A) are routinely referred to other specialists before they see a pediatric rheumatologist [2], at the same time that pediatric rheumatology services are overwhelmed by referrals of children who do not have rheumatic disease [3,4]. This pattern of referral almost assuredly reflects, in part, the fact that joint pain is the most common reason for referrals to pediatric rheumatology clinics and the belief (based on adult models) that joint pain is a symptom of chronic arthritis in children. Work by McGhee and colleagues has shown unequivocally, however, that it is not [5]. In that same study, McGhee and colleagues reported that the second most common reason for referral for pediatric rheumatology consultation was "abnormal laboratory tests," most commonly ANA or rheumatoid factor assays. However, a growing body of evidence accumulated over more than twenty *years *has shown that these tests have limited or no utility in the primary care settings where they are most commonly used.

This short report will summarize some of the evidence base that supports a more limited use of ANA and rheumatoid factor tests by primary care physicians evaluating children for musculoskeletal complaints and/or suspected rheumatic disease. It is intended as a review for primary care physicians who wish to better understand how to use and interpret these two commonly-ordered tests.

Rheumatoid factor (RF) tests remain a standard assay for screening adult patients with musculoskeletal complaints and, depending on the population studied, may be positive in as many as 85% of adults with rheumatoid arthritis and fewer than 10% of healthy adult individuals [6]. This test, now performed using several different methods, detects IgM antibodies directed against the Fc portion of IgG molecules. However, in 1986, Eichenfield and colleagues [7] demonstrated that: [1] most children with positive RF tests don't have JRA/JIA; [2] most children with JRA/JIA don't have positive RF tests; [3] children with JRA/JIA who *did *have positive RF tests could be easily identified on the basis of the history and physical examination; the test did nothing to establish the diagnosis. McGhee and colleagues [5] corroborated this same finding, as shown in Table 1. In that study, 16 children were referred for evaluation of positive rheumatoid factor tests, three of whom (19%) had JRA/JIA. Furthermore, as was reported in the Eichenfield paper, the test added nothing to the history and physical examination, which would have established the diagnosis even if the RF test had been negative.

**Table 1:** Sensitivity of commonly used laboratory tests for diagnosing chronic arthritis in children^#^

**Test cited as reason for referral**	**Number Referred**	**JRA/JIA**	**Other**
IgM-RF	16	3 (19%)*	13 (81%)

ANA	90	14 (16%)	76 (84%)

# – Summary of data from McGhee et al, ref. [5]

Although the prevalence rate of RF-positive JRA/JIA is higher in certain populations (e.g., American Indians [8]), the diagnostic utility of this test is likely to be limited even in high-prevalence populations, owing to the fact that: [1] the prevalence of a positive test in healthy children from the same populations is unknown; and [2] the exuberant nature of the pathologic process in RF-positive children allows the diagnosis to be made on the basis of the history and physical alone, as demonstrated by both the McGhee and Eichenfield groups. Indeed, Eichenfield and colleagues concluded that, "Testing for rheumatoid factor is a poor screening procedure for juvenile rheumatoid arthritis in the general situations in which it is more likely to be requested..." [7]. Given that McGhee and colleagues have corroborated the findings from the 1986 study, it seems just as reasonable to state categorically that *there is never a reason to request a rheumatoid factor assay as a diagnostic test on a child*. Pediatric rheumatologists will, however, continue to use rheumatoid factor testing as a prognostic biomarker until better indicators of prognosis emerge.

Antinuclear antibody (ANA) assays have the opposite shortcoming of RF assays: they are commonly positive in healthy children [9,10]) and the presence of ANA seems to carry no increase in risk for eventually developing rheumatic disease [11]. Furthermore, McGhee and colleagues [12] demonstrated that the titers of ANA seen in children with diverse rheumatic diseases such as JRA/JIA, ankylosing spondylitis, and dermatomyositis, so completely overlap those of healthy children as to make a positive test useless in distinguishing a healthy child from one with any of these diseases. Indeed, ANA tests showed diagnostic utility only in identifying children with systemic lupus erythematosus (SLE), where titers of ≥ 1:1,080 were common. However, even in children with SLE, there remained considerable overlap in their ANA titers (1:360–1:640) with those of otherwise healthy children.

The limits of ANA in evaluating children with suspected JRA/JIA was corroborated in the earlier McGhee study [5], as shown in Table 1. In that study, 90 children were referred to a university-based pediatric rheumatology service because of the results of ANA testing. Only 14 of these children (16%) had JRA/JIA, and, as was seen with rheumatoid factor testing, the ANA test did nothing to support the diagnosis; that is, the diagnosis was already apparent from the history and physical examination and could have been made even if the test were negative. Based on these data, it is reasonable to recommend that ANA be used as a screening test in children only to answer one diagnostic question: *Does this child have systemic lupus? *It would be reasonable to propose, therefore, that a request for ANA testing in a child be accompanied by requests for a complete blood count and differential, urinalysis, and serum C3 and C4 levels, all of which have a high likelihood of being abnormal in childhood-onset systemic lupus [13]. Not surprisingly, given the demographics of systemic lupus, the positive predictive value of an ANA test will be higher in adolescents (where the prevalence of systemic lupus is higher) and lower in pre-pubertal children, where systemic lupus is rare. Indeed, we feel confident in telling the parent of a child 10 years of age or younger with an ANA test of ≤ 1:160 that, "The ANA test was negative."

It should be evident from the foregoing discussion that autoantibody testing has a much more limited utility in the evaluation of children with musculoskeletal complaints or suspected rheumatic disease than it does in adults. This is not to say that the laboratory is not helpful. A complete blood count and differential may, for example, provide the diagnosis in a child with severe musculoskeletal pain and refusal to walk, as musculoskeletal pain, and even frank arthritis, are common presentations of children with leukemia [14,15]. Similarly, in a child with an erythematous, hyperkeratotic rash across her MCP and PIP joints (Gotron's papules), serum levels of CPK and/or aldolase are very likely to reveal that the underlying diagnosis is dermatomyositis [16].

The reader will note that, in each of the above examples, the utility of the laboratory derives from the ability of the clinician to formulate a differential diagnosis based on the history and physical examination. Having a mental category called "rheumatic disease" is not particularly helpful in assessing children, as the different rheumatic diseases display a broad spectrum of presentations, affect children of different ages, and are characterized by physical findings that show only limited overlap between the different disease entities. This means, therefore, that the practitioner needs to be familiar with the *distinctive *clinical presentations of rheumatic disease in children, including the typical signs and symptoms, age range of the affected children, and diseases that mimic those under primary consideration.

It is reasonable for clinicians to desire a simple "test" that will allow them to consider or exclude "rheumatic disease" as a broad category in children. Unfortunately, such a test doesn't exist, any more than there's a "test" that excludes metabolic disease (aminoacidopathies present with a very different clinical picture from that of disorders of glucose metabolism) or endocrine disease (children with defects in steroid synthesis present at different ages and with different symptoms compared with children with type 1 diabetes). There is no reason to believe that such a test will ever emerge. We are therefore left with what good primary care physicians have always relied on: a good history, focused physical examination, and broad knowledge base. After all, *children aren't just adults*.
